# Ethical reasoning through simulation: a phenomenological analysis of student experience

**DOI:** 10.1186/s41077-016-0027-9

**Published:** 2016-08-08

**Authors:** Gareth Lewis, Melissa McCullough, Alexander P Maxwell, Gerard J. Gormley

**Affiliations:** 1 Centre for Medical Education, Queen’s University Belfast, Belfast, Northern Ireland; 2 Department of Clinical Medicine, Brighton and Sussex Medical School, Brighton, UK; 3 Regional Nephrology Unit, Belfast City Hospital, Belfast, Northern Ireland

**Keywords:** Simulation, Ethics, Phenomenology, Professionalism

## Abstract

**Background:**

Medical students transitioning into professional practice feel underprepared to deal with the emotional complexities of real-life ethical situations. Simulation-based learning (SBL) may provide a safe environment for students to probe the boundaries of ethical encounters. Published studies of ethics simulation have not generated sufficiently deep accounts of student experience to inform pedagogy. The aim of this study was to understand students’ *lived experiences* as they engaged with the emotional challenges of managing clinical ethical dilemmas within a SBL environment.

**Methods:**

This qualitative study was underpinned by an interpretivist epistemology. Eight senior medical students participated in an interprofessional ward-based SBL activity incorporating a series of ethically challenging encounters. Each student wore digital video glasses to capture point-of-view (PoV) film footage. Students were interviewed immediately after the simulation and the PoV footage played back to them. Interviews were transcribed verbatim. An interpretative phenomenological approach, using an established template analysis approach, was used to iteratively analyse the data.

**Results:**

Four main themes emerged from the analysis: (1) ‘Authentic on all levels?’, (2)‘Letting the emotions flow’, (3) ‘Ethical alarm bells’ and (4) ‘Voices of children and ghosts’. Students recognised many explicit ethical dilemmas during the SBL activity but had difficulty navigating more subtle ethical and professional boundaries. In emotionally complex situations, instances of moral compromise were observed (such as telling an untruth). Some participants felt unable to raise concerns or challenge unethical behaviour within the scenarios due to prior negative undergraduate experiences.

**Conclusions:**

This study provided deep insights into medical students’ immersive and embodied experiences of ethical reasoning during an authentic SBL activity. By layering on the human dimensions of ethical decision-making, students can understand their personal responses to emotion, complexity and interprofessional working. This could assist them in framing and observing appropriate ethical and professional boundaries and help smooth the transition into clinical practice.

**Electronic supplementary material:**

The online version of this article (doi:10.1186/s41077-016-0027-9) contains supplementary material, which is available to authorized users.

## Background

Medical ethics can be considered as the moral principles that underpin and govern a person’s behaviour and actions in a healthcare setting. Clinicians encounter many ethical dilemmas in practice that require careful management to avoid significant harm occurring to patients, carers and healthcare providers. These ethical issues are particularly acute for medical students in their transition to becoming junior doctors. Ethical dilemmas and the associated interpersonal interactions are cited by junior doctors as key areas of unpreparedness in which they often encounter *moral distress* [1]. This moral distress can occur when a conviction as to what is the ethically correct thing to do is constrained by institutions and structures that prevent realisation of this desired course of action. Over time, this distress can result in moral injury manifesting as psychological and emotional harm suffered when individuals are repeatedly subject to taxing ethical and emotional situations for which they feel unprepared and which go against moral norms or expectations [2].

Traditionally, medical ethics teaching focuses on grounding students in ethical principles and applying these to theoretical cases. The format of such teaching often comprises didactic lectures and seminars with idealised ethics problems [3]. Critically, such teaching methods are not situational and do not afford learners an opportunity to experience the complex social and emotional dimensions that occur in ‘real-life’ ethical dilemmas [4]. For many, the first time encountering the contextual realities of ethical problems will be when they start working as a junior doctor.

### Complexity and the simulation-based education paradigm

In terms of pedagogical frameworks, there is a need to build on work that helps prepare students to navigate and manage ethical quandaries in clinical practice. Simulation-based learning (SBL) methods are widely used to advance students’ clinical skills and behaviours before they transfer into clinical practice. SBL can provide learning opportunities for large cohorts of medical students that are not readily available in workplace environments [5, 6]. Additionally, SBL may offer an immersive and embodied experience that introduces learners to complex ethical dilemmas.

Clinical workplaces are often dynamic and challenging environments in which to work. Often, healthcare professionals will encounter uncertainty, unpredictability and risk whilst carrying out their duties. Exposure to clinical uncertainty in medical curricula may better prepare students to manage complexity and explore the boundaries of professional competence and so improve their preparation for practice [7]. SBL has an established evidence base for developing technical and procedural abilities in idealised settings [8]. The fidelity of SBL in mirroring authentic real-life uncertainty and complexity is much less clearly defined [9–11]. The use of theorised research is thus central in helping to understand and improve such methods of learning in these settings [12].

Few published studies to date have used complex SBL to teach ethics or explore student experience in depth [13–16]. Evidence is emerging that increasing the complexity and realism of such scenarios has a positive effect on learning [17]. Realistic SBL can provide a safe environment for students to develop moral imagination and more effectively engage with ethical dilemmas [18]. Moral imagination is that capacity which allows individuals to identify with others, exercise empathy, imagine future possibilities and consequences as a result of a course of moral action and respond appropriately [18].

Our study aimed to understand the fine-grained nuances of medical students’ lived experiences in coping with the dynamic clinical, social and emotional challenges of managing ethical dilemmas within a SBL environment. We also aimed to gain potential teaching insights.

### Epistemological position and conceptual orientation

This qualitative study was underpinned by an interpretivist epistemology given the nature of the research aims and the complex social dimensions that occur within a high authenticity ward-based simulation teaching activity. Existential phenomenology focuses on the description and interpretation of lived experiences in order to truly understand a phenomenon as it exists in the consciousness of an individual [19]. Therefore, phenomenology was used as a conceptual framework for this study due to its appropriate theoretical fit for examining and interpreting the richness of student experience [20, 21].

## Methods

### Setting and context

The study was performed in Queen’s University Belfast (QUB), Northern Ireland. The medical degree programme at QUB follows a 5-year integrated curricular model, with increasing clinical exposure through years 3 to 5. Students are exposed to SBL activities throughout the entire curriculum.

### Recruitment and sampling

Ethical approval was obtained from the Medical School’s Research Ethics Committee in advance of the study (Ref: 13/36v2), and written consent for participation and use of audio and video recordings was obtained from all participants and simulation scenario actors. Medical students in their fourth year of study were invited by email to participate. A matrix of willing participants and their demographic characteristics was used to select a purposeful sample of subjects to ensure they were representative of the larger year group in terms of gender, age, graduate entry status and whether they were from within or outside the European Union. Phenomenological sampling aims to strike a balance between the far-reaching insights gained by broad sampling of different respondents and the deep understanding of the phenomenon that can be yielded from in-depth analysis. Therefore, sample sizes are generally smaller to permit more thoughtful analysis and not to be overwhelmed by the volume of data [12, 22, 23]. In keeping with other similar phenomenological-based studies, eight participants were therefore recruited for the study [22, 23] (see Table 1).

**Table 1:** Demographic details and pseudonyms of study participants

Pseudonym	Age	Sex (M/F)	Graduate entry (Y/N)	EU citizen (Y/N)
Cheryl	26	F	N	Y
Craig	21	M	N	Y
Duncan	23	M	N	Y
Jaya	22	F	N	N
Mary	22	F	N	Y
Naomi	22	F	N	Y
Phoebe	24	F	Y	Y
Rachel	27	F	Y	Y

### Description of the ward-based SBL activity

An interprofessional SBL ward-based activity was developed by a multidisciplinary team incorporating practitioners from medicine, nursing, ethics and law, psychiatry, drama and simulation, as well as simulated patients and medical students. The aim was to create a naturalistic, immersive environment that allowed participants to have an embodied and reactive learning experience, without risk of patient harm. The SBL setting was framed as a typical hospital ward. Participants were asked to assume the role of a first year junior doctor who had been assigned to this ‘ward’. They were asked to perform a range of clinical duties but were not briefed about the specifics of any ethical issues that would emerge. The ethical dilemmas incorporated into the SBL activity have been identified as key issues for which medical students and junior doctors express the need for more guidance [24]. These included dealing with end-of-life issues, family conflict, breaches of confidentiality, raising concerns about a colleague’s unprofessional behaviour and appropriate use of social media.

Both scenarios (see below) were run simultaneously and adjacent to each other in a bespoke simulated ward environment in the Clinical Skills Education Centre at QUB. The scenarios lasted for up to 15 min. Several individuals wearing scrubs or wearing stethoscopes were present along with simulated patients and relatives. All interacted with each other and the participant in a naturalistic manner as if they were in a busy hospital ward. Realistic medical equipment, including fixtures such as hand-washing stations, patient charts, observation machines, oxygen and IV fluid equipment were all present (see Additional file 1).

#### Scenario 1: end-of-life care and advance directive

The student is a first year doctor working in a medical admission unit. They are asked by the nurse in charge to prescribe analgesia for a patient with end-stage chronic obstructive pulmonary disease. As they approach the patient’s bedside, they are greeted by a person claiming he is the patient’s son. He is domiciled in Canada but has had to return to the UK to see his mother. He discusses the status of an advanced directive signed by the patient and her daughter (his sister) refusing mechanical intubation and cardiopulmonary resuscitation. He found this document in the patient’s medical record and asks for it to be repealed citing concerns over its validity. The student participant is interrupted throughout the scenario by nursing staff asking for completion of discharge documentation for other ward patients. The observation chart records increasing pulse rate, falling blood pressure, rising respiratory rate and low oxygen saturations necessitating consideration for transfer to an intensive care unit. The patient’s daughter then enters and an argument between the son and daughter occurs over whether to resuscitate the mother should she have a cardiorespiratory arrest. Towards the end of the encounter, they both ask the student whether the advance directive can be over-ridden.

#### Scenario 2: maintaining professional boundaries and social media

The student is a first year doctor working in a medical admission unit. A nursing colleague has asked that urinalysis be performed on a patient with Parkinson’s disease and who is known to be verbally inappropriate with female staff. The student, whilst performing and recording urinalysis on a replica sample of urine at the patient’s bedside, is made to feel uncomfortable by over-familiar and mildly flirtatious comments from the simulated patient. After the urinalysis is completed and documented in the simulated medical notes, the student is then met by the nurse who is very friendly and supportive, acknowledging the inappropriateness of the patient. The nurse asks whether the student would like to contribute comments made by the simulated patient to a social media page that the ward staff host. She tells the student it is a bit of “fun”.

A range of reality cues were incorporated into the design of the SBL activity to maximise the authenticity of the exercise [25]. Aside from the key clinical and ethical dilemmas that students encountered, these cues included the following: emotional (*upset patient relatives*), psychological (*cognitive loading with interruptions and multiple competing tasks*), material (*clinical equipment*, *medical charts and professional dress*) and sensory (*ambient ward noise playing in the background*) cues. Simulated patients were guided in their roles and trained using improvisation theatre techniques. This aimed to promote interactive role playing that was both emergent and reactive [26, 27].

### Data capture

**Fig. 1:**
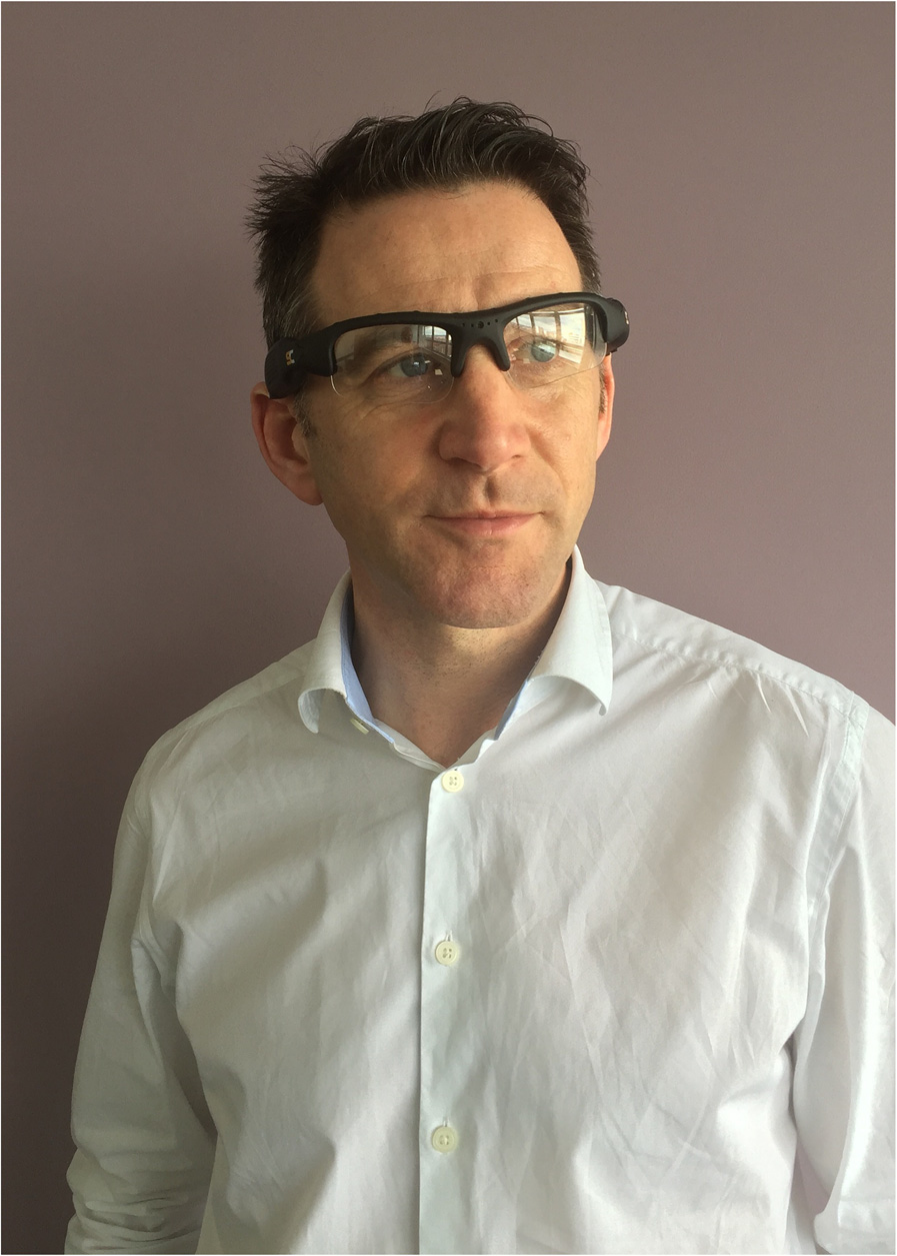
The video camera head glasses were unobtrusive even for participants who wore spectacles underneath

In order to augment data gathering, participants wore digital video glasses (SunnyCam™ HD) to capture point-of-view (PoV) film footage of their SBL experience (see Fig. 1). This footage recorded a *trace* through participants’ experiences in the complex simulation environment. Such video methods of capturing data have been used in a wide range of settings, including healthcare, to capture the perspective of subjects in order to understand more closely their actual experiences [28, 29]. Immediately following the scenarios, participants were interviewed individually by one of two researchers (GL or GG). Interviews were exploratory and open-ended in nature, deriving from the content of what participants were sharing in order to remain as true as possible to their experiences. As the interview proceeded, issues were probed in more depth and reference was made to PoV footage. This helped in unearthing each participant’s embodied experiences in an attempt to uncover perceptual knowledge that usually remains tacit [30]. Interviews were audio-recorded and transcribed verbatim including paralanguage. Participants were assigned pseudonyms (see Table 1) and quotations below use the nomenclature: *I*—investigator, *P*—participant.

### Analysis

A range of qualitative analyses was performed in this project. In this primary analysis, a phenomenological analysis using a template analysis approach was used to analyse the data [22]. A priori areas of interest were used to identify themes expected to be relevant to the analysis a number of weeks before data collection commenced. This promoted reflexivity by bringing to the surface researcher assumptions and presuppositions that were identified and noted. This aimed to mitigate the effects of unconscious bias on the part of researchers during participant interviews, for example, by ensuring that some areas were not favoured or pursued more intensively unless the participant had identified these as significant. Interviews were transcribed verbatim and analysed separately. Themes derived from the first two transcripts were coded independently by the researchers, and each subsequent transcript was analysed without reference to the analysis of previous transcripts. Themes derived from the first two transcripts were entered into an initial template and compared with the a priori areas of interest previously identified. Subsequent themes from the remaining six transcripts were entered into another template which was then applied to the entire dataset. Once the final template was agreed, all transcripts were coded against it and used to illuminate the interpretation of the dataset.

Consistent with a phenomenological methodology, reflexive diaries before, during and after analysis were kept and referred to. As both researchers had been closely involved in the design and running of the SBL scenarios, by fostering dialogue, cross-checking of transcript analysis and discussing complementary and divergent interpretations, hidden beliefs and biases could be surfaced in order to strengthen the validity of the findings.

## Results

Eight participants took part in the study generating 63 min of video footage and 311 min of interview data. Detailed analysis of the verbatim transcripts of these interviews yielded four main themes: (1) ‘Authentic on all levels?’, (2) ‘Letting the emotions flow’, (3) ‘Ethical alarm bells’ and (4) ‘Voices of children and ghosts’. Consistent with a phenomenological approach, we have given equal weight to each participant’s experience and therefore have not quantified in the text the number of participants reporting a particular phenomenon.

### Authentic on all levels?

Whether the SBL was perceived as being authentic varied in time and with situation. Participants recounted the SBL activity as feeling both *true to life*, yet at the same time recognising its artificial nature—‘a nice touch’:

It was the fact that there was other things going on as well there were people walking around you could see, you know you were looking about seeing and noticing that there were different things going on. It wasn’t as if people were just waiting for you to come it felt as though that was a nice touch. [Craig]

Participants toggled between *reality* and *simulation* as the scenario unfolded and situations and emotions became more complex. The tone and paralanguage of those interviewed became more intense and colloquial when describing stressful or emotionally laden moments. During these times in the SBL exercise, participants reminded themselves they were in a simulation in order to control these emotions.

Participants in the SBL environment discussed how they attempted to keep simulation ‘as real as possible’. The researchers’ explicit attempts at creating realism through the use of reality cues, such as being interrupted to complete patient discharge documentation, were recognised as such and paradoxically became fiction cues:

Since I knew it was a simulation I felt it was almost a deliberate distractor so I said to the nurse I would be there in a minute and I would deal with this first. [Duncan]

However, uncertainties in the environment, such as the identity of other individuals, drew participants into an internal dialogue in which they tried to deduce what was happening based on information gathered. Participants reported this as reinforcing their perception of dealing with a true to life situation, even though in their thinking they acknowledged, at points throughout the exercise, its simulated nature.

There were potential real-world implications of making an error in the medication prescription chart (kardex).

**I:** Simulator environment – did you feel safe?

**P:** Yeah

**I:** Did you feel that you could have harmed anybody or did you feel you maybe over-took risks

**P:** I felt as though if I had been left to write the kardex, I mean, I was worried in case I might have made a mistake, and was, like, you know you were aware that ok, it’s simulation but at the same time, you know if I did make a mistake, someone probably would have picked up on it and would have called me up on it and the situation would have changed… [Craig]

Craig illustrates that the SBL experience can be authentic on different levels, but not necessarily at the same time and in the same way. He felt the reality of worrying about the patient safety aspects of an incorrect prescription on the medication chart (kardex). Yet, he gained a measure of psychological safety in knowing the exercise was a simulation. A further psychological safety net is Craig's belief, when imagining (cf ‘at the same time') if this were not a simulation, that a real-life mistake would have been ‘picked up on’. In this case, both his own safety and that of the patient would be maintained.

Alongside participants’ authentic experiences were a range of physiological responses to increased stress. These included ‘warmth’, ‘sweating’, a ‘fight or flight’ response, an ‘adrenaline’ rush, ‘heart going a dinger’ and a distorted perception of time. Some of these were quantitatively expressed with resolution of difficulties leading to a ‘dipping’ in anxiety ‘levels’. Despite uncertainties and anxieties in the scenarios, all participants felt safe to explore the boundaries of ethical practice in the SBL setting. Whilst participants recognised the potential real-world implications of error, being able to recall this was a simulation provided a psychological safety net that errors would not result in actual harm. Additionally, though participants had been briefed that they could withdraw from the SBL exercise at any time, none chose to exercise this option. Whilst participants felt ‘pushed’, none were pressed beyond their tolerable limits. All commented that they would like to re-engage with similar SBL scenarios at a later date, articulating a greater confidence in handling them.

### Letting the emotions flow

Emotions featured prominently in participants’ interviews, and each recalled unpleasant negative feelings such as fear, anxiety, awkwardness, loneliness and regret, through to embarrassment and feeling silly. Less often, more positive feelings of humour and relief were present. All participants had to navigate a rapidly shifting emotional landscape as the scenario unfolded.

Strategies for dealing with intense high-arousal emotions by suppression and refocusing were described. For example, allowances were made for a patient’s inappropriate remarks by rationalising these as arising from his medical condition or cultural background. Sublimation of strong emotion into concentrating on the correct performance of a technical task helped maintain control. The emotional colouring of a situation could subtly vary, and this is instanced in the desire of participants to escape from the arguing relatives during the end-of-life scenario. An initial response of ‘getting out of there to get help’ becomes a desire to leave to find a consultant *in order* to then return with that senior presence to achieve emotional resolution. Participants were able to refashion their primitive visceral responses—the flight response of ‘getting out of there’, into constructive strategies—leaving to find help. Despite incomplete resolution of an ethical dilemma, Duncan experienced a sense of emotional fulfilment when some of the underlying tension was defused. He had done all he could to try and resolve a disagreement between a patient’s relatives. Even though there was no complete resolution, he had reassured them, and he viewed this positively:

At this point I was actually feeling pretty good em, I felt at the end there was a bit of resolution to the scenario…obviously they were still arguing when I left, but I felt that I had reassured them both and that there was a clear plan from here…I did feel a sense of closure and did feel satisfied… [Duncan]

The role of previous life experiences assisted in real-time processing of feelings. Drawing on similar experiences of encountering inappropriate behaviour, participants were primed with a repertoire of coping strategies such as maintaining distance and being ‘very professional, nice and pleasant’. This permitted them to regulate their feelings of awkwardness.

### Ethical alarm bells

Participants readily perceived the explicit ethical dilemmas relating to autonomy and confidentiality present within the two SBL scenarios. Recognising the implicit ethical considerations for truth-telling and raising concerns when confronted with an unprofessional nursing colleague in the ‘maintaining professional boundaries’ scenario was more challenging for some. Of interest were further, emergent ethical dimensions. A urinalysis specimen bottle was unintentionally mislabelled during one iteration of the above scenario. This was recognised by the participant who resolutely refused to accept the validity of the results and insisted on such to the simulated nurse.

‘Alarm bells’ rang when ethical boundaries were crossed. Trigger phrases such as ‘Facebook’, or obvious transgressions of confidentiality in both scenarios, empowered the students to take action. Robust *moral courage* was displayed when refusing to give credence to results from incorrectly labelled specimens despite repeated reassurance or persistent ‘seductive’ attempts to solicit patient information for uploading to Facebook. Moral courage is ‘the individual’s capacity to overcome fear and stand up for his or her core values’ [31]. It is the willingness to speak out and do which is right in the face of forces that would lead a person to act in some other way. Such moral courage was displayed by taking refuge in the tenets of ethical principles:

…. that was my only way to get out of this was to throw in that word and hope she doesn’t push on further after that cos I would just have to keep repeating patient-doctor confidentiality, patient-doctor confidentiality. [Jaya]

There was less clarity when other ethical boundaries were approached. The specific legal status of the advance directive in the ‘end-of-life care’ scenario was unknown by participants. Duncan felt an unease ‘at the back of [his] mind’ when discussing confidential patient information with a person who ‘appeared to be a nice guy’ at the relative’s bedside. The smiling and supportive nurse made it more difficult for participants to challenge her unprofessional behaviour. Cheryl tells a falsehood about not having a Facebook page when asked by the nurse, describing subsequent regret she told a lie when viewing the PoV footage (see Additional file 2). Others displayed avoidant behaviour by not rebutting the nurse’s insistence that it was ‘ok’ to post patient-identifiable comments on social media.

The approach of these ethical boundaries could be masked by ignorance of the legislative backgrounds in one case and the context-dependent nature in which they arose. In the latter cases, a friendly person or smiling face disguised the request to compromise confidentiality and subsequently made it more difficult for participants to challenge unprofessional behaviour.

### Voices of children and ghosts

Participants used the phrase ‘first day’ when describing the progression from medical student to a Foundation Programme Year 1 (F1) doctor. Accompanying the transition into this new environment and culture are residual tensions and concerns derived from undergraduate experience. Some felt unable to raise concerns or make suggestions due to their inexperience. Students have an insubstantial presence on the wards; they feel like ‘ghosts’; they cannot fully enter into what is happening and ‘soak up the anxiety’. They are like ‘little children who should be seen and not heard’. They are a nuisance or inconvenience if they did speak up; senior staff have ‘a right’ to get annoyed with junior staff by virtue of their position on the medical hierarchy.

Em, cos, well I know I was being an F1 but as a medical student whenever I go to the hospitals it always feels like, we’re in the way of doctors and nurses like, this is their job whereas we are technically still not qualified and so even if we see something that’s not right or what not like I feel we can’t really voice our opinions, cos if we do we’ll be like those whistleblowers kind of thing and then they’ll be thinking like we’re just medical students how can you pass these comments, you know we’ve been working here for years and you know what’s right kind of stuff, so that’s why I always feel inferior to doctors and the nurses working, and I know it’s wrong and I should probably just voice it out saying I’m going to be part of the team but I guess because of their years of experience wise I always just tend to clam up and not say anything. [Jaya]

Participants described anxiety at the presence of a consultant in the scenario. This inhibited their performance of the urinalysis as they were worried about being asked something they did not know. Descriptions of ward attachments in which the participants suggested some areas for improvement that were not taken seriously reinforced a passive and disempowering stance towards the workplace. These feelings of passivity spill over into how interprofessional relationships with nursing staff are viewed. Nursing staff have to be ‘appeased’, they can ‘make or break’ a junior doctor’s working life and one has to be ‘careful’ when dealing with the nurses. An example of nursing staff gossiping about another student went unaddressed by one participant who avoided challenging or reporting unprofessional behaviour. Another recounted being shouted at by a theatre nurse and the negative effect this had on their views of nurses more generally.

## Discussion

This study provided an opportunity to gain a deeper insight into the lived experiences of students as they encounter ethical dilemmas in an immersive SBL environment. From a practitioner’s perspective, dealing with real-life ethical dilemmas is often challenging, particularly for junior doctors in their transition from medical school. Aside from the application of ethical principles that underpins such decision-making, the unavoidable human and emotional dimensions that are evoked by such dilemmas are integral to their resolution and require a degree of moral imagination [18]. Strategies to develop moral imagination—the ability to respond to others empathically, envisage future states through a process of internal dramatic rehearsal and the use of metaphors to understand ethical situations, are thus critically important to nurture in medical training [32]. Using a SBL exercise to immerse students in a complex ethical dilemma may be one such strategy. Critical to immersion is the emotions that follow an experience. Emotions were not passive passengers for participants on their SBL journey but active drivers that shaped and nuanced specific solutions. The video glasses provided PoV video footage that enriched specific learning without reducing immersion; participants described they had largely forgotten about wearing them by the end of the exercise.

### Authenticity unlocking learning spaces

SBL in which a highly faithful representation of an ideal procedure or scenario exists is useful in the development of basic technical skills. It could be assumed therefore that SBL activities will only achieve their full pedagogical potential when all components are as lifelike as possible. However, this may not necessarily be the case. Whilst it is evident that SBL experiences should not be overly simplistic, reductionist or predictable, recreating closely a complex, real-life ethical dilemma may not require all parts to be fully authentic at all times and in all ways for participants. Reality and fiction cues in SBL may not be perceived in the same way by participants [33]. In our study, inserting interruptions to mimic reality during the end-of-life care scenario actually reduced immersion for Duncan. The real requirement in this SBL exercise was to create authenticity in the uncertain ethical complexities faced by participants whilst ensuring that the supporting environment and actors did not overly detract from immersion or the perception of authenticity.

When learners are placed in an authentic real-life situation potentially demanding many competencies to be exercised, those areas in which the learner feels weaker may well cause the greatest anxiety for him or her. This was observed in the present study where, early on in these scenarios, it became clear where the fault lines lay for participants. Some were confident with technical tasks, others less so. A minority felt able and relished the challenge to engage with the human characters in the SBL activity whereas others on seeing nursing or senior medical staff immediately became suspicious or fearful. The value of these authentic and complex SBL environments is that learners’ particular issues and concerns readily rise to the surface as they face the complexity encountered by clinical practitioners. This permits more targeted and student-centred learning. For example, the specific issues uppermost in the minds of each student could be readily elicited during debrief interviews. The PoV footage unlocked aspects of student experience that were previously hidden behind layers of memory and the differences between recall versus actual experience [34]. The PoV footage captured Phoebe describing herself as being ‘overwhelmed’ immediately after the scenario. Throughout the interview, she had neither stated nor hinted at this. In phenomenological terms, by reviewing the footage, the ‘onion skin layers’ overlying her significant lived experience were peeled away [19]. The contours of her actual experience, rather than her mediated external projection of it, were exposed. In Phoebe’s case, the authentic emotions created by the SBL unlocked real learning; despite feeling ‘overwhelmed’, she did not feel able to reveal this to others.

### Opening up the emotional dimension for learners

Emotions play an important role in the learning process as well as in the development of interpersonal skills and medical professionalism [35]. Nevertheless, the measurement, teaching and assessment of emotional awareness and intelligence in medical school curricula remain controversial and in need of further research [36]. In McNaughton’s work on how emotion is conceptualised within medical education, she presents three discourses on emotion: emotion as physiology, emotions as skills and emotion as a socio-cultural mediator [37]. Results in our study indicate that SBL can evoke emotion in all three domains described by McNaughton. This provides an opportunity to set our work within this theoretical framework and so to understand and harness the influence of emotion on the clinical learning environment. Participants described a broad range of emotion with associated physiological and autonomic accompaniments. The additional effects on cognition and behaviour reinforced to participants that what they were feeling was very real. Emotion as skills was demonstrated in the way some participants wanted to escape from a difficult situation and how they then rationalised and sublimated these initial feelings into providing patient-centred care. The third domain of emotion as socio-cultural mediator is appreciated when considering the SBL activity as furnishing students with a transformative learning experience. Here, they develop a deeper empathy and a greater emotional resilience and are changed in a foundational way by their experiences. This may assist learners in bridging the gap observed in the literature between stated moral intentions and actual moral actions in an emotionally fraught environment [38, 39]. By revealing those factors that lead to personal, ethical or moral compromise, learners may be more able to gain control of them on subsequent occasions.

### Moral distress, courage and compromise in ethically complex narratives

Both moral distress and moral courage are attended by strong emotional colouring that can profoundly influence ethical decision-making [37, 40]. However, the influence of emotions on clinical professional development, learning processes and ethical decision-making remain substantially under-investigated.

In this present study, instances of both moral courage (refusal to credit the validity of an incorrectly labelled specimen) as well as moral compromise (telling an untruth about not having a Facebook page) were evident. These were nuanced by the particular emotional and professional contexts in which these occurred [41]. The clinical necessity of analysing a correct specimen superseded the reassurances from the simulated nurse. On another occasion, the friendliness and perceived emotional safety afforded by this same nurse after a difficult patient encounter weakened moral resolve to tell the truth and challenge unprofessional behaviour.

Significantly, participants viewed their situation regarding raising concerns when on clinical placements in diminutive and insubstantial terms—‘little children’ and ‘ghosts’. They expressed feeling unable to challenge the status quo and voice legitimate concerns. This is most concerning, in light of the findings from healthcare inquiries such as the Francis Report [42], that whilst trainees should be the ‘invaluable eyes and ears in a hospital setting’, there is for some an acclimatisation during undergraduate training to the acceptance of poor practice and unprofessional behaviour and a learned helplessness in being able to challenge this. Furthermore, participants described a seamless transition of interprofessional caricatures and prejudices into the SBL environment. Students default to the position that the presence of senior staff is stressful and uncomfortable and threatens to expose their ignorance. Nursing staff are viewed with suspicion. This ‘learning’ from previous clinical attachments went unquestioned and unanalysed. A hidden curriculum of negative interprofessional interactions and subsequent paralysis in raising concerns was brought to the surface by the SBL activity. What was previously latent became open to critique and modification.

#### Strengths, limitations and future directions

The key strengths of this study were twofold; it examined a critical area of clinical practice for which junior doctors feel unprepared and employed interview and data capture methods that unearthed deep student experiences that have so far been relatively opaque to investigation. Limitations of this work include the extent of its generalisability to other healthcare settings. Given the theoretical and epistemological orientation of this study, generalisability was not an intended aim. Rather, it was to demonstrate the transferability and value of the approach in exposing these depths of student experience.

We aimed in this study to unearth the nuances of individual experience and so provide truly valuable learning that was personally transformative for that student. Participants had a window opened into their deep personal behaviours and reactions during an authentic clinical simulation that exposed potential fault lines in the foundation of their evolving professionalism. These included hierarchical power gradients, interprofessional tensions and a negative culture in the workplace concerning raising concerns. Left unaddressed, these threaten effective, transparent team-working and ultimately patient safety. By employing SBL methods, possibilities to strengthen and build up the foundations of ethical and professional resilience become apparent. Training students to recognise and refashion feelings and behaviours in difficult interprofessional situations may maximise rich team learning.

The use of high-authenticity SBL in helping students to better navigate ethical encounters requires evidence of effectiveness. We would argue this is a fruitful line of inquiry for research into the usefulness and practicality of SBL in developing wider ethical, moral and professional capabilities.

## Conclusions

This work demonstrates the powerful synergy of an authentic clinical environment, an emotionally diverse landscape and an ethically complex narrative in surfacing rich and specific learning for each individual student. A safe pedagogical space is created for learners to explore and recognise the limits of their capabilities. They can begin to frame professional boundaries and behaviours in line with professional codes of conduct. In terms of curricular development, simulation of ethical dilemmas in a complex environment may provide an important bridge for students as they transition into professional practice from medical school. Such simulations can layer on many of the human dimensions of applying ethical principles in practice and aid in the delivery of truly patient-centred care. SBL employing ethical and emotional complexity could be an important tool to expose and address the hidden curricula that follow the undergraduate journey. In doing so, it is hoped that students will become more deeply reflective and resilient doctors.

## Abbreviations

F1, Foundation Programme Year 1; QUB, Queen’s University Belfast; SBL, simulation-based learning

## Additional files

Additional file 1: Simulated ward environment in which the end of life scenario unfolded. This footage was taken from a pilot study to assess the feasibility of the SBL activity set-up. In this clip, the PoV footage was captured by a BulletCam mounted via a Velcro fastening to the side of participants’ heads. Because of superior alignment, sound and video capture quality, the SunnyCam glasses were employed in the scenarios described in this present study. Available from: https://www.dropbox.com/s/t866g7ihxzfatuk/Additional%20File%201.avi?dl=0. (AVI 91.6 mb)

Additional file 2: PoV footage. This participant (Cheryl) tells a falsehood to the nurse during the maintaining professional boundaries scenario about not having a Facebook page. She regretted telling a lie when later viewing this footage. Available from: https://www.dropbox.com/s/cd944ht8irfj5pa/Additional%20File%202.wmv?dl=0. (WMV 45.7 mb)

## References

[1] Monrouxe LV, Bullock A, Rees C, Mattick K, Webb K, Lall K, Lundin R (2015). Foundation doctors, transitions and emotions.

[2] Howe A, Smajdor A, Stöckl A (2012). Towards an understanding of resilience and its relevance to medical training. Med Educ.

[3] O’Sullivan H, Van Mook W, Fewtrell R, Wass V (2012). Integrating professionalism into the curriculum: AMEE guide no. 61. Med Teach.

[4] Cordingley L, Hyde C, Peters S, Vernon B, Bundy C (2007). Undergraduate medical students’ exposure to clinical ethics: a challenge to the development of professional behaviours?. Med Educ.

[5] Khan K, Pattison T, Sherwood M (2011). Simulation in medical education. Med Teach.

[6] Singer BD, Corbridge TC, Schroedl CJ, Wilcox JE, Cohen ER, Mcgaghie WC, Wayne DB (2013). First-year residents outperform third-year residents after simulation-based education in critical care medicine. Simul Healthc.

[7] Tallentire VR, Smith SE, Skinner J, Cameron HS (2011). Understanding the behaviour of newly qualified doctors in acute care contexts. Med Educ.

[8] Morgan PJ, Cleave-Hogg D, Desousa S, Lam-McCullough J (2006). Applying theory to practice in undergraduate education using high fidelity simulation. Med Teach.

[9] Curtis MT, Diazgranados D, Feldman M (2012). Judicious use of simulation technology in continuing medical education. J Contin Educ Health Prof.

[10] Norman G, Dore K, Grierson L (2012). The minimal relationship between simulation fidelity and transfer of learning. Med Educ.

[11] Bland AJ, Topping A, Tobbell J (2014). Time to unravel the conceptual confusion of authenticity and fidelity and their contribution to learning within simulation-based nurse education. A discussion paper. Nurse Educ Today.

[12] Cook DA, Hamstra SJ, Brydges R, Zendejas B, Szostek JH, Wang AT, Erwin PJ, Hatala R (2013). Comparative effectiveness of instructional design features in simulation-based education: systematic review and meta-analysis. Med Teach.

[13] Gisondi MA, Smith-Coggins R, Harter PM, Soltysik RC, Yarnold PR (2004). Assessment of resident professionalism using high-fidelity simulation of ethical dilemmas. Acad Emerg Med.

[14] Brondani MA, Rossoff LP (2010). The “hot seat” experience: a multifaceted approach to the teaching of ethics in a dental curriculum. J Dent Educ.

[15] Smith KV, Witt J, Klaassen J, Zimmerman C, Cheng AL (2012). High-fidelity simulation and legal/ethical concepts: a transformational learning experience. Nurs Ethics.

[16] Smith KV, Klaassen J, Zimmerman C, Cheng AL (2013). The evolution of a high-fidelity patient simulation learning experience to teach legal and ethical issues. J Prof Nurs.

[17] Gillan PC, Jeong S, van der Riet PJ (2014). End of life care simulation: a review of the literature. Nurs Educ Today.

[18] Chen RP (2011). Moral imagination in simulation-based communication skills training. Nurs Ethics.

[19] Smith JA, Flowers P, Larkin M (2009). Interpretative phenomenological analysis: theory, method and research.

[20] Greenfield B, Jensen GM (2010). Beyond a code of ethics: phenomenological ethics for everyday practice. Physiother Res Int.

[21] Greenfield B, Jensen GM (2010). Understanding the lived experiences of patients: application of a phenomenological approach to ethics. Phys Ther.

[22] King N, Cassell C, Symon G (2004). Using interviews in qualitative research. Essential guide to qualitative methods in organizational research.

[23] Mclachlan E, King N, Wenger E, Dornan T (2012). Phenomenological analysis of patient experiences of medical student teaching encounters. Med Educ.

[24] Asghari F, Samadi A, Rashidian A (2013). Medical ethics course for undergraduate medical students: a needs assessment study. J Med Ethics Hist Med.

[25] Gormley G, Sterling M, Menary A, Mckeown G (2012). Keeping it real! Enhancing realism in standardised patient OSCE stations. Clin Teach.

[26] McCullough M (2012). Bringing drama into medical education. Lancet.

[27] Rossiter K (2012). Bearing response-ability: theatre, ethics and medical education. Journal of Medical Humanities.

[28] Lahlou S (2011). How can we capture the subject’s perspective? An evidence-based approach for the social scientist. Soc Sci Inform.

[29] Lahlou S, Le Bellu S, Boesen-Mariani S (2015). Subjective evidence based ethnography: method and applications. Integr Psychol Behav Sci.

[30] Gore G, Rix-Lièvre G, Wathelet O, Cazemajou A, Skinner J (2012). Eliciting the tacit: interviewing to understand bodily experience. The interview: an ethnographic approach.

[31] Lachman VD (2007). Moral courage: a virtue in need of development?. Medsurg Nurs.

[32] Coeckelbergh M, Mesman J (2007). With hope and imagination: imaginative moral decision-making in neonatal intensive care units. Ethic Theory Moral Prac.

[33] Dieckmann P, Manser T, Wehner T, Rall M (2007). Reality and fiction cues in medical patient simulation: an interview study with anesthesiologists. J Cogn Eng Decis Mak.

[34] Fourie MM, Kilchenmann N, Malcolm-Smith S, Thomas KGF (2012). Real-time elicitation of moral emotions using a prejudice paradigm. Front Psychol.

[35] McConnell MM, Eva KW (2012). The role of emotion in the learning and transfer of clinical skills and knowledge. Acad Med.

[36] Cherry MG, Fletcher I, O’Sullivan H, Dornan T (2014). Emotional intelligence in medical education: a critical review. Med Educ.

[37] Mcnaughton N (2013). Discourse(s) of emotion within medical education: the ever-present absence. Med Educ.

[38] Feldmanhall O, Mobbs D, Evans D, Hiscox L, Navrady L, Dalgleish T (2012). What we say and what we do: the relationship between real and hypothetical moral choices. Cognition.

[39] Navarrete CD, McDonald MM, Mott ML, Asher B (2012). Virtual morality: emotion and action in a simulated three-dimensional “trolley problem”. Emotion.

[40] Leblanc VR, McConnell MM, Monteiro SD (2015). Predictable chaos: a review of the effects of emotions on attention, memory and decision making. Adv Health Sci Educ Theory Pract.

[41] Greene JD, Nystrom LE, Engell AD, Darley JM, Cohen JD (2004). The neural bases of cognitive conflict and control in moral judgment. Neuron.

[42] Department of Health (2013). The Mid Staffordshire NHS Foundation Trust Public Inquiry, Chaired by Robert Francis QC. (Francis report).

